# Suboptimal Linkage to Care of Delta-Infected Patients in an Area with Increasing Migration-Driven Prevalence of Hepatitis D in Recent Years

**DOI:** 10.3390/v18020174

**Published:** 2026-01-28

**Authors:** Ângela Carvalho-Gomes, Ariadna Bono, Lola Gómez, Susana Sabater, Juan Carlos Rodríguez, Antonio Palau, Ana Forés, María Rodríguez, Sonia Pascual, Maria Àngels Cebrià i Iranzo, Martín Prieto, Marina Berenguer

**Affiliations:** 1 Hepatology, Hepatobiliopancreatic Surgery and Transplant Group, La Fe Health Research Institute (IIS La Fe), 46026 Valencia, Spainangeles.cebria@uv.es (M.À.C.i.I.);; 2 Centro de Investigación Biomédica en Red de Enfermedades Hepáticas y Digestivas (CIBERehd), Instituto de Salud Carlos III (ISCIII), 28029 Madrid, Spain; 3 Hepatology Unit, Department of Gastroenterology, La Fe University Hospital, 46026 Valencia, Spain; 4 Department of Microbiology, La Fe University and Polytechnic Hospital, 46026 Valencia, Spain; 5 Castellón General University Hospital, 12004 Castellón, Spain; 6 Dr. Balmis General University Hospital, 03010 Alicante, Spain; rodriguez_juadia@gva.es (J.C.R.);; 7 Alicante Institute for Health and Biomedical Research (ISABIAL), 03010 Alicante, Spain; 8 Physiotherapy Department, University of Valencia, 46010 Valencia, Spain; 9 Medicine Department, University of Valencia, 46010 Valencia, Spain

**Keywords:** hepatitis delta, linkage to care, epidemiology, migration

## Abstract

**Background and Aims**: Changes in hepatitis delta virus (HDV) epidemiology have been highlighted recently in the context of increasing worldwide migrations. The lack of comprehensive real-world data on HDV in the Valencia region highlights the need for a structured registry to accurately estimate disease prevalence and burden and to generate robust real-world evidence on clinical outcomes and therapeutic effectiveness. We aimed to better understand the barriers for successful HDV patient care in our region by establishing a registry as well as linking previously under-recognized or lost to follow-up (FU)cases to care. **Methods**: After a search of all possible HDV cases in a Spanish region, attempts were made (through letters and phone calls) to relink to care those lost to FU. Two approaches were undertaken: (i) search of the Microbiology Labs Database, and (ii) clinical chart review from adult patients attending the Hepatology or Infectious Disease (ID) Units outpatient clinics of the three participant hospitals between January 2011 and June 2021. **Results**: Only one third of anti-HDV positive patients without adequate clinical management could be successfully linked or re-linked to care, highlighting a substantial gap in follow-up. Among 243 HDV cases detected (7.5% of HBsAg-positive patients), 111 belonged to the hospitals’ health department, and after excluding deceased or transplanted individuals, the final study cohort consisted of 84 patients. Of these, 27.4% were adequately followed in Hepatology or Infectious Disease Clinics, 11.9% had been inadequately followed recently, 45.2% had been lost to follow-up for several years, and 15.5% had never been evaluated in outpatient clinics. Overall, only a third of the patients without adequate clinical management could be successfully linked/relinked to care. **Conclusions**: In our setting, only a minority of anti-HDV positive patients are adequately managed in specialized outpatient clinics, with unsuccessful attempts to link many patients to care, particularly among young migrant men. These findings underscore the need for alternative strategies, such as decentralized testing, reflex testing, and the involvement of patient navigators or social workers, to strengthen linkage to care and improve retention.

## 1. Introduction

Chronic hepatitis D (CHD), also known as Delta hepatitis, is a rare infectious disease with a globally variable and likely underestimated prevalence. Current estimates suggest that between 12 and over 60 million people may be infected worldwide, reflecting substantial underdiagnosis. The distribution is uneven, with higher prevalence in endemic regions such as the Mediterranean basin [1,2,3]. Numerous studies have indicated that hepatitis D virus (HDV) coinfection accelerates and intensifies the progression of liver disease compared to hepatitis B virus (HBV) mono-infection [1,4] leading eventually to higher mid-term mortality in HBV/HDV infected patients [1,5].

For many years, the typical HDV patient profile in Spain was that of a middle-aged man, born in Spain, with a history of intravenous drug use (IDU) as the primary risk factor [6]. Until 2020, treatment options for CHD were limited to the off-label use of pegylated interferon alfa (PEG-IFN-α) in highly selected patients. Results with PEG-IFN-α were generally unsatisfactory, with only a quarter of patients achieving HDV RNA negativity at the end of therapy, and half of these experiencing relapses after drug discontinuation [7,8]. Among the new drugs under evaluation, Bulevirtide (BLV, Hepcludex^®^), an HBV/HDV entry inhibitor [9,10,11,12], received conditional marketing authorization from the European Medicines Agency (EMA) in June 2020 for chronic hepatitis delta with compensated liver disease and a positive HDV RNA viral load [13]. Since its approval, an increasing body of real-world evidence has emerged regarding the use of BLV, predominantly in European patients with advanced fibrosis or cirrhosis. The effectiveness observed in real-life settings is consistent with that reported in clinical trials, with a substantial proportion of patients achieving marked reductions in HDV RNA levels and normalization of ALT after several months of therapy, along with a favorable safety profile [11]. Like the approach with hepatitis C, the identification of HBV/HDV cases becomes crucial once effective drugs are developed to enhance treatment efficacy, mitigate long-term consequences, and prevent local transmission. Recommended strategies for HDV detection/screening from the 2023 European Association for the Study of the Liver (EASL) guidelines are to test at least once all HBsAg-positive individuals for anti-HDV reflex testing whenever clinically indicated (acute decompensation, aminotransferase flares) or yearly in those remaining at risk of infection [14]. Anti-HDV tests should be followed by HDV RNA determination in all anti-HDV-positive individuals double reflex testing [15]. However, despite these recommendations, adherence to universal HDV screening remains suboptimal across Europe. In contrast, the American Association for the study of Liver Diseases (AASLD) advocates anti-HDV testing only in high-risk HBsAg-positive individuals (i.e., IDU history, men who have sex with men, individuals at risk of acquiring sexually transmitted diseases, and migrants from areas where HDV is endemic) [16,17].

The first epidemiological study in our region in almost thirty years was published in 2020 by Hernàndez-Èvole et al. [18]. The study reported on clinical, serological, analytical, and histological data from HDV-infected patients in a reference Hepatology Unit of a large reference Hospital, confirming a more aggressive course of HBV/HDV coinfection compared to HBV mono-infection. Notably, a discernible shift was observed in patient demographics, with an increasing prevalence among Eastern European migrants over the last few years. The study also highlighted a relative lack of awareness of this infection and its potential consequences in recent years, leading to delayed diagnoses. Altogether, the above findings underscore the need for thorough screening of HDV infection, particularly in HBsAg-positive patients belonging to at-risk populations.

The identified trends prompted us to adopt a more ambitious approach, expanding the study to cover a larger geographical area. In addition, the COVID-19 pandemic substantially disrupted chronic hepatitis care, resulting in delayed monitoring and treatment and leaving a proportion of patients lost to follow-up, as reported in multiple studies, with potential implications for long-term liver outcomes [19,20]. Our specific objectives were threefold: (i) to establish a registry of HDV-infected patients in our region; (ii) to determine the proportion of under-recognized hepatitis delta cases within the chronically infected HBV patient population; and (iii) to facilitate the linkage to care of previously under-recognized and/or lost to follow-up (FU) patients.

## 2. Patients and Methods

This is a multicenter retrospective study with a prospective phase conducted in 3 healthcare departments in the Valencian Community, Spain. Each department is served by a specific university reference hospital in each of the three provinces in which the Valencian Community, one of the seventeen autonomous regions of Spain, is divided as follows: La Fe University Hospital in Valencia, General University Hospital in Castellón, and Dr. Balmis General University Hospital in Alicante. These three healthcare departments are part of the Public Healthcare System of the Valencian Community, a network of 24 healthcare departments that provides universal free healthcare coverage to approximately 95% of the inhabitants in the Valencian Community. Two out of the three study centers had implemented Reflex testing since 2005 and 2013, respectively. One of the centers had not implemented reflex testing at the time of the study due to administrative constraints. The study protocol was approved on 7 March 2022 by the Ethics Committee of Clinical Research of La Fe Universitari and Politécnic Hospital (IIS La Fe CEIM: ref number: IN-ES-980-6335) and was conducted in accordance with the Helsinki Declaration of 1975. Individuals younger than 18 years were excluded from participation. Identifiable information was not collected; all data were accessible solely to the research team and used strictly for research and clinical purposes. The exemption from informed consent was authorized due to the nature of the procedure, the guidelines followed subsequently corresponding to standard clinical practice in such cases.

This study was divided into two phases. In the first phase, a case detection approach was employed using two methods: (a) prospectively detecting HDV cases from HBsAg-positive samples through a search of the Microbiology Labs Database in each participating hospital (using Clinisys™ GestLab laboratory information system (LIS), Tucson, AZ, USA); (b) retrospectively detecting cases from electronic medical records of adult patients who attended the outpatient clinics of the three hospitals between January 2011 and June 2021 (using the Orion Clinic clinical-care information system with keywords related to delta hepatitis). The keywords used for the search were as follows: delta chronic hepatitis, delta hepatitis, HDV hepatitis, delta virus hepatitis; HBV-HDV hepatitis, chronic HBV hepatitis, chronic hepatitis B, hepatitis B, hepatitis B, HBV, HDV, hepatitis delta virus, hepatitis B virus, acute hepatitis B, acute HBV hepatitis. This methodology aligns with the approach taken in the preliminary study [18].

The second phase involves a prospective FU of diagnosed HDV-infected patients aiming to establish linkage to care of those lost to FU or those who had never attended Hepatology or Infectious Disease Units outpatient clinics. The administrative databases of the hospitals were searched for information on home addresses and phone numbers. For individuals lost to FU or who had never attended, multiple attempts to contact them were made through letters and phone calls (up to six attempts at different time points, spaced one to two weeks apart). The personnel responsible for patient contact were proficient in five languages, while written correspondence was sent exclusively in Spanish. For migrants without contact information in the hospital database, non-governmental organizations (NGOs) were contacted to encourage patients to attend the hospital and complete the required tests, to refer them to the hospital, and to provide their contact information for clinical follow-up.

Patients were categorized as follows: (i) part of the Spanish healthcare system and adequately followed in the Hepatology or ID Units outpatient clinics based on the American Association for the Study of Liver Diseases (AASLD) and European Association for the Study of the Liver (EASL) guidelines recommendations with at least one annual visit; (ii) part of the Spanish healthcare system but inadequately followed since 2020 due to COVID healthcare disruptions; (iii) part of the Spanish healthcare system but lost to follow-up, after a variable duration of time attending the Hepatology or ID outpatient clinics, due to various reasons; and (iv) never attended or evaluated in a Hepatology or ID Unit outpatient clinic.

HBsAg-positive patients with serum samples lacking HDV serology results over the past 10 years were contacted for the determination of anti-HDV antibodies (IgG+IgM assessed by EIA/CIA using the ETI-AB-DELTAK-2 kit from DiaSorin (Stillwater, MN, USA)). In cases where anti-HDV was positive, quantification of HDV RNA was carried out using the RNART.HDV.CE and DRNA.CE kits from DiaPro (Sesto San Giovanni, Italy) (sensitivity has varied from 1000 copies/mL to 150 copies/mL) depending on the date of detection (1000 copies/mL until 2023). HDV RNA quantification was performed for all linked patients with a detection limit of 150 copies/mL. For non-linked patients, HDV RNA was measured using the assay available at the time, with a detection limit of 1000 copies/mL, which may have led to misclassification of low-level viremia as negative. Reanalysis of these samples was not feasible, as specimens collected before 2022 are no longer available due to the hospital’s sample storage policy.

Data on epidemiology, virology, histology, and clinical aspects, including therapeutic details, severity of the underlying disease, comorbidities, and outcome variables, were gathered. In cases where information from the prior 3 years was unavailable, elastography and/or other non-invasive methods (FIB-4 and APRI scores) were employed to determine the stage of fibrosis.

Patient monitoring and treatment adherence to the recommendations provided by EASL and/or AASLD were collected.

Based on (i) previous epidemiological data indicating a prevalence of HBV infection in Spain at around 0.6–1% [18] and (ii) a collective service area of the three study hospitals covering approximately 400,000 inhabitants within the Valencian Community (https://lafe.san.gva.es/ca/; http://castellon.san.gva.es/; https://alicante.san.gva.es/ca/, accessed on 21 July 2021), we expected a cohort size of 2500 to 4000 HBV-infected individuals, with 250 to 400 of them potentially infected with the delta virus.

The assessment of HDV-infected patients included the following aspects: (i) prevalence across different participant groups (patients, migrants, and data from the bibliography); (ii) demographic and epidemiological data (age, gender distribution, country of origin, and number of subjects); (iii) FU data, including the stage of the disease. Numerical data is presented as means with standard deviations or medians with interquartile ranges, as appropriate, while categorical data is expressed as counts and percentages. The normality of data distribution was evaluated using the Kolmogorov–Smirnov test. Statistical analyses were conducted both numerically and graphically, employing standard methods such as chi-square tests, *t*-student, ANOVA, Mann–Whitney U, or Kruskal–Wallis, as appropriate. Multiple logistic regression analyses were used to assess the factors independently associated with loss to FU or successful re-linkage. A *p*-value < 0.05 was considered statistically significant, and a Bonferroni correction was applied to all reported *p*-values. The statistical analysis was performed using the SPSS program version 25.0 (IBM corporation, Armonk, NY, USA).

## 3. Results

### 3.1. Anti-HDV Patient Population

**Figure 1:**
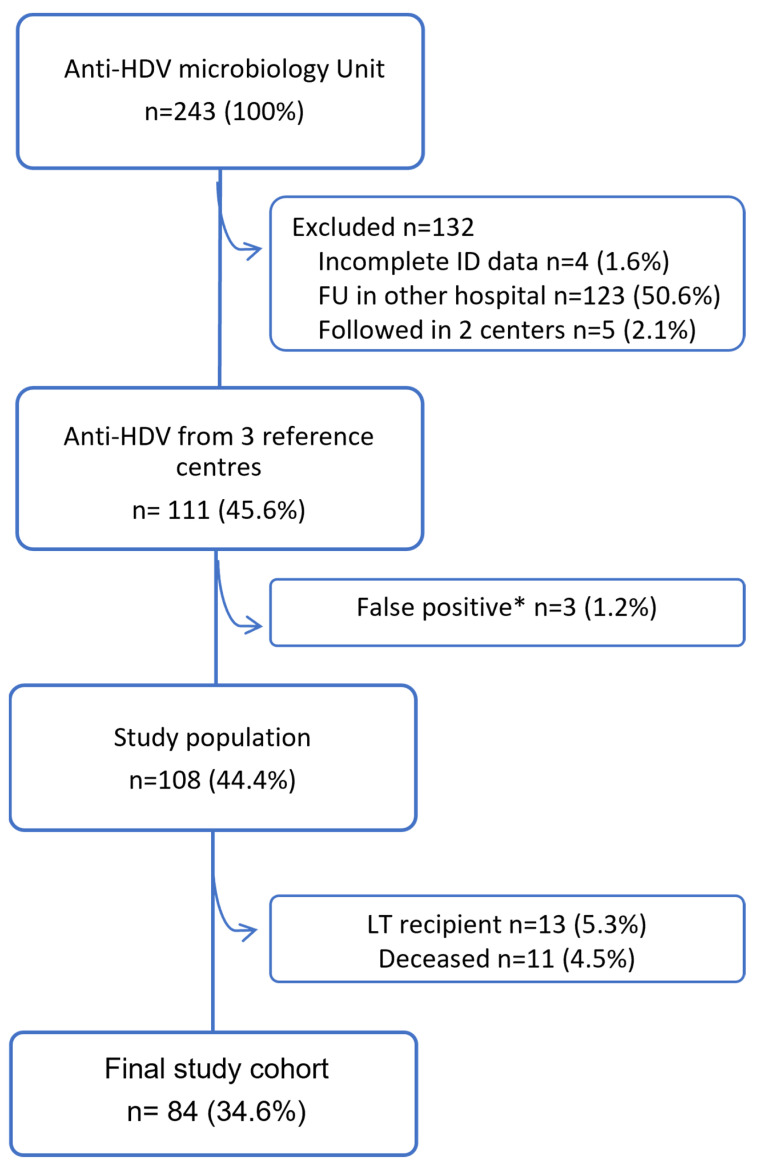
Flow-chart of patient selection. FU, follow-up; HDV, hepatitis delta virus; ID, identification; LT, liver transplantation. * False positives due to laboratory errors.

Two hundred forty-three anti-HDV cases were detected (7.5% of the HBV patients) (Table 1). Of these, only 111 patients belonged to the three the hospital’s health departments (45.7%) (Figure 1) after excluding 4 patients with incomplete ID data, 5 patients followed at the same time in two of the 3 centers, and 123 with FU in different healthcare departments.

After reviewing the charts of these 111 patients, we excluded 3 false anti-HDV positive cases (only one positive result followed by >5 negative), such that the study population was represented by 108 HDV cases (6.2% of the HBsAg (+) belonging to the Hospital health department), with the highest prevalence in the health department of Castellón (14.5%), followed by Valencia (5.6%) and Alicante (3.3%) (Table 1) (Castellón vs. Valencia *p* < 0.0001, Castellón vs. Alicante *p* < 0.0001, Valencia vs. Alicante *p* = 0.0328).

Of the 108 delta cases, 13 had undergone liver transplantation (LT), and 11 had died, 5 due to liver-related causes. Of the remaining 84 patients, which comprised the final study cohort, HDV RNA had been assessed in only 48 patients (57.1%) at either diagnosis or follow-up, with viremia detectable in only 15 (17.9%). Comparison of patient subgroups according to whether HDV RNA testing was conducted revealed statistically significant differences in terms of history of IDU, HIV co-infection, and cirrhosis. These differences may reflect the large number of unknown or missing values in the group without HDV RNA testing, due to either inadequate or no FU (Supplementary Table S1).

Of the 84 patients, only 23 (27.38%) were adequately being followed in the Hepatology or ID outpatient clinics, 10 (11.90%) had been recently inadequately followed in the context of COVID-related healthcare disruption –“COVID-disrupted”, 38 (45.24%) were patients that had been lost to FU for several years and the remainder 13 (15.48%) anti-HDV positive cases had never been evaluated in a Hepatology/ID clinic (Table 2). Baseline characteristics of patients adequately followed vs. those either never evaluated or inadequately followed in the context of COVID-related health care disruption/lost to FU are shown in Table 3. The prevalence of cirrhosis (47.8% vs. 16.4%, *p* = 0.005) and the proportion of patients with HDV RNA viremia assessment (100% vs. 41.0%, *p* < 0.001) were significantly higher in patients with adequate follow-up. Notably, no significant association was observed between country of birth and adequacy of follow-up (*p* = 0.625).

A more detailed analysis of the patients without adequate clinical management is shown in Table 4, and reasons for absence/inadequate FU are described. Most were middle-aged men, with a significant proportion not born in Spain, except for the group where care was recently disrupted by COVID.

Excluding the 10 patients with COVID-related healthcare disruption, a variety of reasons accounted for the absence of adequate FU in the remaining 51 patients, with change in address (23.5%) and admission to a penitentiary center (17.6%) being the most commonly reported. In most instances, though, the reason was unknown due to the impossibility of reaching out to individuals after several attempts by different means (letter, phone calls) (51.0%).

### 3.2. Linking/Relinking to Care

Overall, only 31.1% (19 out of 61) of patients without adequate clinical management were linked/relinked to care (Table 1). Compared to those who could not be linked/relinked, those successfully linked/relinked were more often women (47.37 vs. 19.05, *p* = 0.02) and more often born in Spain (63.2% vs. 35.7%, *p* = 0.04) (Table 5).

More specifically, of the 13 individuals with a positive anti-HDV antibody test who had never attended a liver clinic, only 4 (30.8%) were linked to care, all of whom were foreign-born individuals. After initial linking, one of these patients stopped coming to the hospital and was again lost to FU. Of the 38 lost to follow-up, only 5 (13.2%) were relinked to care, including 4 of 22 Europeans and 1 of 14 Africans (no data on race for two additional persons) (Table 5). Lastly, all 10 patients (100%) were adequately monitored until COVID irruption resumed adequate FU during the study period.

Of all patients successfully linked/relinked to care, only five (26.3%) had positive HDV RNA on linkage to care. Cirrhosis was present in three (15.8%), although none was decompensated, all ultimately developed hepatocellular carcinoma (HCC). The mean time from HDV diagnosis to HCC detection was 7.7 ± 4.0 years. One of the patients died after linkage to care due to respiratory infection in the setting of HCC therapy, and another due to non-related liver disease (Table 5).

In multivariate analysis (Table 6), absence of cirrhosis was independently associated with loss to follow-up (OR: 0.249; 95% CI: 0.074–0.841; *p* = 0.025), while HCV coinfection was significantly associated with successful re-linkage among patients without adequate follow-up (OR: 20.525; 95% CI: 1.323–318.422; *p* = 0.031). Although not statistically significant, a trend toward higher successful re-linkage rates was observed among patients born in Spain compared with migrants (OR: 0.118; 95% CI: 0.013–1.044; *p* = 0.055).

## 4. Discussion

In the current landscape of advanced therapies and evolving epidemiology, there is a need for a comprehensive search and phenotyping of HDV infection cases. A preliminary analysis reported in 2020 [18] revealed a shift in the epidemiology of HDV infection in our setting. Most new diagnoses are now occurring in migrants from endemic areas, often with delayed diagnoses when the disease has already progressed to advanced stages of fibrosis.

Moreover, some HBV-infected patients have unfortunately never been tested for HDV infection [21]. Our hypothesis was that overlooked HBV/HDV coinfection is more widespread than previously estimated, potentially due to rising migration from HDV endemic areas [22]. The changing HDV-infected patient profile suggests that coinfected individuals are predominantly men, migrants from Eastern Europe, with advanced fibrosis and frequent coexistence of cofactors associated with liver disease [18].

This study was designed to enhance our understanding of the local epidemiology of HDV infection, delineate the current phenotype of HDV-infected patients, and establish a connection to care for those previously undiagnosed or lost to FU.

To identify HDV-infected patients across three reference centers in a Spanish region, all individuals with chronic HBV infection recorded in Microbiology Laboratory databases and followed in Hepatology and Infectious Diseases outpatient clinics were systematically reviewed to ensure that anti-HDV testing had been performed at least once within the previous decade. Using this approach, 6.2% of HBV patients were found to be anti-HDV positive, with higher prevalence in a center serving a large migrant population from Eastern Europe. This aligns with European studies reporting increased HDV prevalence among migrants from historically endemic regions, highlighting the need to incorporate migration patterns into screening strategies and micro-elimination initiatives [23].

Reflex testing for HDV was implemented in two of the three participating centers in 2005 and 2013, respectively, whereas in the third center, HDV diagnosis required additional testing. These findings support current recommendations from international and national scientific societies, including the Spanish Association for the Study of the Liver, advocating systematic reflex testing as a critical entry point into the HDV cascade of care [14,16]. However, consistent with previous cascade-of-care analyses in viral hepatitis, reflex testing alone proved insufficient to prevent downstream attrition. Notably, even in centers with long-standing reflex testing, up to 42.6% of diagnosed patients were lost to follow-up, highlighting that improvements in case detection must be accompanied by effective linkage and retention strategies [24].

Most anti-HDV positive patients were middle-aged men, not born in Spain (53.6%), with only 27.4% of them being adequately followed in specialized outpatient clinics. The lack of a statistically significant association between country of birth and adequacy of follow-up (*p* = 0.625) constitutes a notable negative finding. Similar observations have been reported in European viral hepatitis cohorts, where after adjustment for demographic and clinical variables, country of birth did not independently influence critical outcomes such as all-cause or liver-related mortality [25,26].

Approximately 12% of the patients had been adequately monitored until the pandemic disrupted their follow-up, and all of these were successfully relinked to care. However, nearly two-thirds had either never been evaluated in a specialized clinic or had been completely lost to FU for several years. These findings reveal substantial challenges in HDV management and are consistent with previous reports highlighting limited awareness of HDV among both patients and healthcare providers [27,28]. Insufficient understanding of disease severity, the need for long-term monitoring, and gaps in provider knowledge regarding HDV diagnosis and management likely contribute to this high proportion of inadequately followed individuals.

Overall, our results align with the growing micro-elimination literature emphasizing that structural and social determinants—particularly those affecting migrant populations—play a major role in losses across the HDV care cascade [24,29]. Targeted interventions, including active recall systems, patient navigation, culturally adapted models of care, and educational initiatives for both patients and healthcare professionals, are therefore essential to translate improved HDV case identification into sustained engagement in care and meaningful reductions in HDV-related morbidity.

Regrettably, just 31.2% of anti-HDV–positive patients were successfully linked or re-linked to care, a rate consistent with European HCV studies reporting 20–45% re-linkage using active strategies [24,30,31]. These patients, predominantly from Eastern Europe, often presented with advanced liver disease, including cirrhosis or HCC—notably, two of the three patients who were HDV RNA-negative at linkage—although none exhibited decompensated cirrhosis at the time of evaluation. Among the five viremic patients who were re-linked, only one received Bulevirtide; three did not meet current national criteria for treatment, and one was lost to follow-up again.

Although only a minority of re-linked patients had detectable HDV RNA at the time of linkage, this finding is clinically and epidemiologically significant. Individuals with detectable HDV RNA represent a continued source of viral transmission and constitute a subgroup at particularly high risk for adverse clinical outcomes. These results underscore the importance of linkage and re-linkage strategies, not only for individual patient management but also for controlling transmission and preventing HCC, particularly in populations with historically limited engagement in care.

Despite not being statistically significant, a trend of higher successful re-linkage rates was observed among patients born in Spain compared with migrants (*p* = 0.055), which may have reached significance in larger cohorts (*n* = 19). This aligns with prior studies showing that migrants face barriers to healthcare access, resulting in lower linkage-to-care success compared with native-born populations [32,33]. These findings highlight the need for culturally and structurally tailored strategies to improve retention in viral care cascades.

Reasons for unsuccessful linkage included imprisonment, change in address, severe comorbidities, or inability to contact patients, predominantly young non-Spanish-born men. Several factors may have contributed to the unsuccessful linkage to care observed in this patient population. Although no formal analytical evaluation was conducted, the experience gained from attempts to re-link patients with this condition allows several relevant observations to be highlighted. Written communication was largely ineffective, whereas direct telephone contact proved more successful. Collaboration with non-governmental organizations facilitated access to translators, reduced language barriers, and enhanced patient trust. Offering same-day diagnostic and clinical appointments improved assessment, though these measures alone were insufficient. Cultural and language barriers, as well as limited understanding of the healthcare system, likely further hindered effective communication and continuity of care.

Overall, limited awareness of HDV remains a significant challenge. Public health initiatives, similar to those implemented for HIV, are needed to increase knowledge about the disease, the importance of follow-up, and strategies to achieve viral elimination. This study has several limitations. Reaching and re-linking vulnerable populations, such as homeless individuals and non-regular migrants, remains challenging. Reflex testing, implemented in only two of the three centers, is considered the most effective method for identifying HDV-infected patients; however, even with automated notification systems, social determinants of health continue to impede successful linkage and management [15]. HDV RNA quantification in the three hospitals did not meet WHO international standards until 2023 (1000 copies/mL versus 75 IU/mL), potentially under-detecting cases before that year. Missing RNA data in 42.9% of patients further limits prevalence estimates. Hidden mortality bias and the small sample size (*n* = 84) also reduce statistical power for subgroup analyses.

Strengths of the study include its multicenter design, active interventions to re-link patients (e.g., phone calls), and real-world data that extend beyond clinical trials. Comparing our loss-to-follow-up rates with European cohorts shows similar patterns. Italian [34] and Spanish [24] studies report substantial attrition along the hepatitis care cascade, while international data [35,36] highlight barriers from limited testing and unmet patient needs. These findings reinforce the importance of structured interventions, including patient navigators and active recall systems, to improve linkage, retention, and treatment uptake in vulnerable populations.

In summary, in a region historically considered at risk, approximately 6% of HBV patients are anti-HDV positive, a figure that has remained stable over time, with 55.6% corresponding to non-Spanish-born individuals. Only one-third of these anti-HDV-positive patients are adequately managed in specialized clinics, and re-linkage remains challenging, particularly among young migrant men. This finding revealed challenges similar to those reported for HCV in Madrid [37], suggesting that barriers to care in this population are systemic and not disease-specific. Other studies from other European countries show comparable patterns [23,38], reinforcing the broader relevance of our findings and the need for structured interventions, such as patient navigators, to improve linkage, retention, and treatment uptake in vulnerable populations.

## Figures and Tables

**Table 1:** Number of positive HBsAg and anti-HDV antibodies tested overall and belonging to the Hospital health department.

	General H (Castellón)	La Fe H (Valencia)	General H (Alicante)	Total
HBsAg (+) (*n*)	511	1683	1038	3232
Anti-HDV (+) (*n*)	53	133	57	243
Proportion of anti-HDV (+)/HBsAg (+) population (%)	10.4%	7.9%	5.5%	7.5%
HBsAg (+) belonging to the Hospital health department (*n*)	282	779	692	1753
Anti-HDV (+) belonging to the Hospital health department (*n*)	41	46	24	111
Confirmed anti-HDV (+) belonging to the Hospital health department (*n*)	41	44	23	108
Proportion of anti-HDV (+)/HBsAg (+) population belonging to the Hospital health department (%)	14.5%	5.6%	3.3%	6.2%
Confirmed anti-HDV (+) of the final study cohort	33	32	19	84
HDV RNA (+) of the final study cohort (*n* = 84) (*n*, %)	8 (24.2%)	5 (15.6%)	2 (10.5%)	15 (17.9%)

**Table 2:** Follow-up status and linkage to care in the final study cohort.

Follow-Up Status	Final Study Cohort *n* = 84	Linked to Care *n* = 19
Adequate follow-up, *n* (%)	23 (27.4%)	Not applicable
COVID-disrupted follow-up, *n* (%)	10 (11.9%)	10 (100%)
Lost to follow-up, *n* (%)	38 (45.2%)	5 (13.2%)
No follow-up, *n* (%)	13 (15.5%)	4 (30.8%)

**Table 3:** Demographic characteristics of patients adequately followed vs. those inadequately followed, either due to recent COVID-related disruption, lost to follow-up, or never attended a specialized clinic.

	Final Cohort Study (*n* = 84)	Adequately Followed (*n* = 23)	Not Adequately Followed (*n* = 61)	*p* Value
Age, years (mean ± SD)	52.2 ± 11.1	55.1 ± 12.7	51.1 ± 12.3	0.192
Gender, male (*n*, %)	58 (69.0%)	14 (60.9%)	44 (72.1%)	0.428
Country of birth (Spain/Other)	39 (46.4%)/45 (53.6)	12 (52.2%)/11 (47.8%)	27(44.3%)/34 (55.7%)	0.625
IDU history	21 (25%)	5 (21.7%)	16 (26.2%)	0.782
HCV infection	23 (27.4%)	5 (21.7%)	18 (29.5%)	0.589
HIV infection	12 (14.3%)	3 (13.0%)	9 (14.8%)	1
Cirrhosis	21 (25%)	11 (47.8%)	10 (16.4%)	0.005
HDV RNA determination	48 (57.1%)	23 (100%)	25 (41.0%)	<0.001
HDV RNA (+)	15 (17.9%)	9 (39.1%)	6 (9.8%)	0.207

NOTE: Missing data were handled using listwise deletion. Abbreviations: IDU, intravenous drug use.

**Table 4:** Demographic characteristics of the patients in need of linking/relinking (inadequate FU due to COVID-related healthcare disruption—COVID-disrupted, lost to follow-up, or never attended in a specialized clinic).

	COVID-Disrupted ^a^ *n* = 10	Lost to FU ^b^ *n* = 38	No FU ^c^ *n* = 13	*p* Value
Age, years (mean ± SD)	59.3 ± 12.7	48.1 ± 12.4	53.5 ± 11.9	0.012 0.002 ^a vs. b^ 0.294 ^a vs. c^ 0.112 ^b vs. c^
Gender, male (*n*, %)	6 (60.0%)	29 (76.3%)	9 (69.2%)	0.572
Continent of birth (*n*, %)				0.119
**-** Europe	10 (100%)	22 (57.9%)	9 (69.2%)	
----Spain	8 (80.0%)	14 (36.8%)	5 (38.5%)	
----Eastern Europe	2 (20.0%)	8 (21.1%)	4 (30.8%)	
**-** Africa	0	14 (36.8%)	3 (23.1%)	
**-** Asia	0	0	1 (7.7%)	
**-** Unknown	0	2 (5.3%)	0	
Country of birth (Spain/Other)	8 (80%)/2 (20%)	14 (36.8%)/24 (63.2%)	5 (38.5%)/8 (61.5%)	0.045
Reason for inadequate FU (*n*, %)				<0.001
**-** serious Comorbidities	0	1 (2.6%)	1 (7.7%)	
**-** Admission to the Penitentiary center	0	8 (21.1%)	1 (7.7%)	
**-** Change in address	0	9 (23.7%)	3 (23.1%)	
**-** Failure to keep medical appointments	0	0	1 (7.7%)	
**-** COVID-associated healthcare disruption	10 (100%)	0	1 (7.7%)	
**-** Unknown	0	20 (52.6%)	6 (46.2%)	

FU: follow-up.

**Table 5:** Clinical features of patients successfully linked/relinked to care.

	Total Linked/Relinked *n* = 19	Relinked COVID-Related Inadequate Monitored Patients *n* = 10	Relinked Lost to FU Patients *n* = 5	Linkage of Previously Unmonitored Patients *n* = 4 *
Age, years (mean ± SD)	55.5 ± 10.9	59.3 ± 11.6	52.6 ± 8.2	49.5 ± 10.7
Gender, male (*n*, %)	10 (52.6%)	6 (60.0%)	2 (40.0%)	2 (50.0%)
Country of birth (Spain/Other)	12 (63.2%)/7 (36.8%)	8 (80.0%)/2 (20.0%)	4 (80.0%)/1 (20.0%)	0 (0%)/4 (100%)
Last FU status (*n*, %)				
FS > 9 Kpa **	3 (15.8%)	3 (30.0%)	0	0
Compensated cirrhosis	3 (15.8%)	3 (30.0%)	0	0
Decompensated cirrhosis	0	0	0	0
HCC	3 (15.8%)	3 *** (33.3%)	0	0
HDV RNA (+)	5 (26.3%)	1 (11.1%)	3 (50%)	1 (25%)
Bulevirtide treatment	1 (5.26%)	1 (10.0%)	0	0
Dead	2 (5.3%)	2 (20.0%)	0	0

* One of these patients was reached and linked to care, but unfortunately restarted abusive alcohol intake and was again lost to FU. ** Determined by transient elastography according to EASL 2017 Clinical Practice Guidelines on the management of hepatitis B virus infection criteria. *** Time from initial HDV diagnosis to HCC diagnosis was 10, 10, and 3 years.

**Table 6:** Multivariate analysis of factors associated with loss to FU or successful re-linkage.

	Loss to FU		Successful Re-Linkage	
Variables	OR [95% CI]	*p* Value	OR [CI]	*p* Value
Age	0.951 [0.896–1.010]	0.100	0.947 [0.872–1.028]	0.196
Gender	1.453 [0.396–5.337]	0.574	1.978 [0.364–10.739]	0.430
Country of birth	1.272 [0.317–5.103]	0.734	0.118 [0.013–1.044]	0.055
HCV Infection	1.611 [0.304–8.550]	0.576	20.525 [1.323–318.422]	0.031
HIV infection	1.858 [0.295–11.717]	0.509	0.203 [0.015–2.793]	0.233
Cirrhosis	0.249 [0.074–0.841]	0.025	2.088 [0.247–17.629]	0.499

Note: Level of significance, *p*-value < 0.05. Abbreviations: FU, follow-up; CI, confidence Interval; OR, odds ratio.

## Data Availability

The data supporting this study are available from the corresponding author upon reasonable request.

## Supplementary Materials

The following supporting information can be downloaded at: https://www.mdpi.com/article/10.3390/v18020174/s1, Table S1: Demographic characteristics of patients with versus without HDV RNA determination.

## Abbreviations

AASLD	American Association for the Study of Liver Diseases
APRI	Aspartate Aminotransferase to Platelet Ratio Index (APRI) scores
CHD	Chronic hepatitis D
EASL	European Association for the Study of the Liver
EIA/CIA	Enzyme Immunoassay/Chemiluminescent Immunoassay
FIB-4	Fibrosis-4 Index
FU	follow-up
HBsAg	hepatitis B virus surface antigen
HBV	hepatitis B virus
HCC	hepatocellular carcinoma
HDV	hepatitis delta virus
ID	Infectious Disease
IDU	intravenous drug use
LIS	laboratory information system
LT	Liver transplantation
NGOs	non-governmental organizations
OR	Odds ratio
RNA	ribonucleic acid
