# Supplementary material for: Suboptimal Linkage to Care of Delta-Infected Patients in an Area with Increasing Migration-Driven Prevalence of Hepatitis D in Recent Years
Source: Viruses. 2026 Jan 28;18(2):174. doi: 10.3390/v18020174 (PMC12945175; doi:10.3390/v18020174)
Supplement: Supplementary file 1 [file viruses-18-00174-s001.zip › viruses-4023284-supplementary.pdf]

Supplementary Table S1. Demographic characteristics of patients with versus without HDV RNA determination.

	HDV RNA determination (n=48)	No HDV RNA determination (n=36)	P value
Age, years (mean ± SD)	51.73 ±11.1	52.8 ± 11.3	0.663
Gender, male (n, %)	32 (66.7%)	26 (72.2%)	0.586
Country of birth (Spain / Other)	22 (45.8%)/17 (47.2%)	17 (47.2%) / 19 (52.8%)	0.899
IDU history (Yes/No/ND)	11 (22.9%)/ 36(75%)/1 (2.1%)	10 (27.8%)/16 (44.4%)/10 (27.8%)	0.001
HCV infection (Yes/No/ND)	11 (22.9%)/37 (77.1%)/0 (0%)	12 (33.3%)/23 (63.9%)/1 (2.8%)	0.266
HIV infection (Yes/No/ND)	6 (12.5%)/42 (87.5%)/0(0%)	6 (16.7%)/25 (69.4%)/5 (13.9%)	0.021
Cirrhosis (Yes/No/ND)	16 (33.3%)/31 (64.6%)/1 (2.1%)	5 (13.9%)/16 (44.4%)/15 (41.7%)	<0.001

Abbreviations: IDU, intravenous drug use
